# Literature Review of Research on Chronic Pain and Yoga in Military Populations

**DOI:** 10.3390/medicines4030064

**Published:** 2017-09-01

**Authors:** Shari Miller, Susan Gaylord, Alex Buben, Carrie Brintz, Kristine Rae Olmsted, Nakisa Asefnia, Michael Bartoszek

**Affiliations:** 1 RTI International, 3040 East Cornwallis Drive, Durham, NC 27709, USA; abuben@rti.org (A.B.); krolmsted@rti.org (K.R.O.); 2 Program on Integrative Medicine, Department of Physical Medicine and Rehabilitation, School of Medicine, CB #7200, University of North Carolina, Chapel Hill, NC 27599, USA; gaylords@med.unc.edu (S.G.); carrie_brintz@med.unc.edu (C.B.); 3 Department of Psychology, University of South Carolina, Barnwell College, P. O. box 124, Columbia, SC 29208, USA; nasefnia@email.sc.edu; 4 Womack Army Medical Center, 2817 Reilly Road, Fort Bragg, NC 28310, USA; michael.w.bartoszek.civ@mail.mil

**Keywords:** chronic pain, military, yoga

## Abstract

**Background:** Although yoga is increasingly being provided to active duty soldiers and veterans, studies with military populations are limited and effects on chronic pain are largely unknown. We reviewed the existing body of literature and provide recommendations for future research. **Methods:** We conducted a literature review of electronic databases (PubMed, PsychINFO, Web of Science, Science Citation Index Expanded, Social Sciences Citation Index, Conference Proceedings Citation Index—Science, and Conference Proceedings Citation Index—Social Science & Humanities). The studies were reviewed for characteristics such as mean age of participants, sample size, yoga type, and study design. Only peer-reviewed studies were included in the review. **Results:** The search yielded only six studies that examined pain as an outcome of yoga for military populations. With one exception, studies were with veteran populations. Only one study was conducted with Operation Enduring Freedom (OEF) or Operation Iraqi Freedom (OIF) veterans. One study was a randomized controlled trial (RCT). Four of the five studies remaining used pre/post design, while the last study used a post-only design. **Conclusions:** Studies on the use of yoga to treat chronic pain in military populations are in their infancy. Methodological weaknesses include small sample sizes, a lack of studies with key groups (active duty, OEF/IEF veterans), and use of single group uncontrolled designs (pre/post; post only) for all but one study. Future research is needed to address these methodological limitations and build on this small body of literature.

## 1. Introduction

Rates of chronic pain in the military are alarmingly high, ranging from 25% to 82% among active duty and veteran populations [1,2,3,4,5]. Data on veterans from the nationally representative National Health Interview Survey reveal high rates: 65.5% reported being in pain. Particularly concerning is that 9.1% of veterans reported severe pain [6]. The prevalence of severe pain was striking for younger veterans who served in Operation Enduring Freedom and Operation Iraqi Freedom (OEF/OIF) in Afghanistan and Iraq. Active duty service members also experience high rates of chronic pain; one study reported a prevalence of 44% after combat deployment [5].

Complicating treatment of chronic pain is its co-occurrence with posttraumatic stress disorder (PTSD) [4,7,8,9,10]. Estimates of co-occurring chronic pain and PSTD range from 10% to 97% [11,12,13,14]. This large variation likely reflects the study population. Rates tend to be lower in samples of patients being treated for chronic pain and higher in studies of people being treated for PTSD. Individuals with co-occurring chronic pain and PTSD experience more intense pain, greater affective distress, and higher rates of disability [9]. Yoga has been studied as a treatment strategy for active duty service members and veterans who have PTSD. In one study of female veterans with a history of sexual trauma, iRest (yoga nidra) was effective in decreasing trauma symptoms and depression [15]. Another study among veterans with military-related PSTD found that yoga was effective in decreasing PTSD hyperarousal symptoms and improving sleep quality [16]. In a study of deployed military personnel, yoga was found to reduce symptoms of combat stress [17].

Opioids are a more traditional approach to treat chronic pain. A meta-analysis of 41 randomized controlled trials (RCTs) found that opioids were more effective than placebos for both pain and functional outcomes [18]. However, effectiveness may not be sustained [19,20]. In addition, patients may experience side effects, the most common being constipation and nausea [21]. Of serious concern is the significant misuse of opioids, which increases the risk of overdose and addiction [20]. A 2008 Department of Defense (DoD) survey among active duty service members found an alarming increase in opioid misuse. Rates doubled from 2002 to 2005 and nearly tripled from 2005 to 2008, with 13.4% reporting misuse in the past month and 17.2% in the past year [22]. One study found that 15% of previously deployed service members who reported experiencing chronic pain also reported opioid use [5]. In addition, 44% of service members using opioids reported either no or mild pain in the last month, although opioids should be prescribed only for moderate to severe pain [23,24]. Fatalities from opioid overdose are rising, and these fatalities can be linked to the misprescribing of opioids [25]. Rates of opioid use are especially high in those with chronic pain and PTSD. One study examined medical records of 141,029 veterans who had served in Iraq and Afghanistan and who had a pain diagnosis within a year of entering the Department of Veterans Affairs health care system. Compared with those without a mental health disorder, veterans with pain and comorbid PTSD were more likely to receive higher-dose opioids, receive two or more opioids concurrently, and obtain early opioid refills [26]. Such data underscore the critical need for nonpharmacological interventions, such as yoga, to reduce chronic pain. 

The growth of yoga in military and veteran settings coincides with an increasing acceptance of complementary and integrative health (CIH) approaches to treat chronic pain among these populations. After the Office of the Army Surgeon General’s Pain Management Task Force released its recommendations in 2010 [27], the DoD began to embrace and promote evidence-based CIH methods for pain management. The DoD was a key member of the National Institutes of Health’s (NIH) Interagency Pain Research Coordinating Committee and informed the development of NIH’s 2016 National Pain Strategy [28]. The report echoed many Pain Management Task Force recommendations, advocating for a realignment of pain management priorities to incorporate CIH modalities such as yoga that addresss the biopsychosocial characteristics of chronic pain [28]. 

The services available to veterans and active duty military personnel seeking non-opioid alternatives to chronic pain treatment have since expanded. From 2011 to 2015, the number of Veterans Health Administration (VHA) facilities that either offered or were in the process of developing at least one CIH modality increased by 8.7%. In addition, the number of facilities specifically offering yoga for managing chronic pain conditions such as back pain, headaches, and fibromyalgia more than doubled [29]. Yoga has been integrated into the military’s evolving model of pain management, and yoga classes can now be found on military installations, at military treatment and VHA facilities, and in the Army’s Wounded Warrior Program’s Warrior Transition Units [30]. 

Military personnel are reportedly very open to the use of CIH approaches. In one study of 401 veterans, 82% had used at least one CIH, and 99% were open to their use [31]. However, although the availability of yoga has increased, utilization of these services remains low. One study found that 36% of active duty personnel reported using at least one CIH modality in the past 12 months, yet only 6.8% reported using yoga or some other form of movement therapy during that same span [32]. Even as yoga classes have become more common at VHA facilities targeting chronic pain management, only a small number of veterans use these services [29]. This stagnation in the use of yoga suggests a need to revise not only the content of the military’s pain management model, but also the way in which that model is promoted to veterans and active duty soldiers. 

Given the military’s promotion of yoga to treat chronic pain, research is needed to test its effectiveness among active duty service members and veterans. To date, a review has not been completed of the effectiveness of yoga on pain in military populations. Such a review is needed to inform future research.

## 2. Methods

We conducted a literature review of the following electronic databases through April 2017: PubMed, PsychINFO, Web of Science, Science Citation Index Expanded, Social Sciences Citation Index, Conference Proceedings Citation Index—Science, and Conference Proceedings Citation Index—Social Science & Humanities. The search included a range of studies, from single group observations to randomized controlled trials. We used the following search terms: yoga, military, veteran, active duty, and chronic pain. Inclusion criteria included instruction of yoga, use of quantitative measures that assessed the effect of yoga on pain, and publication in a peer-reviewed journal. Studies that included only qualitative data were excluded. In addition, we focused our review on yoga studies that included movement, and excluded studies that focused only on breathing exercises, including mindful breathing. We recognize that other types of yoga, such as Sudarshan yoga, consist of controlled breathing techniques [33]. We also excluded yoga nidra, or iRest [34], from this review. Yoga nidra consists of simple breathing exercises and noticing, without attempting to change, different parts of the body; and choosing a sankalpa—a phrase or short sentence of a goal or intention to manifest in one’s life [35]. More than 50 veterans’ hospitals and military installations across the United States are implementing iRest [36], although there are limited studies of iRest in military populations.

## 3. Results

Table 1 and Table 2 delineate the variables that were extracted from each of the studies: demographics (participant age, sex, race/ethnicity, marital status, and employment status), sample size, yoga (content, duration, and frequency of sessions), and methods and results (study design, dependent variables, and general findings). This search yielded six studies that fit our criteria. Although our primary focus was on military populations with chronic pain, we broadened our search to include studies that were conducted in military populations who did not necessarily have chronic pain but that did measure pain outcomes. 

The tables summarize the studies; we delineate the results, strengths, and weaknesses of each study in this section. Three of the publications are by Groessl and colleagues [37,38,42]. Two of the papers are from the same study, with a greater number of enrollees over time. The 2008 paper [37], which used a single-group, pre/post design, reported on the effect of Anusara yoga on pain, depression, fatigue, and quality of life (mental and physical) among veterans experiencing chronic low back pain. Significant decreases were seen in pain as well as in depression and fatigue. The study is notable in its inclusion of measures of depression, fatigue, and quality of life - domains that are related to chronic pain. Groessl’s second study [38] builds on the first with an expanded sample of subsequent enrollees. The study examined gender differences in the effects of the Anusara yoga program. As compared to men, women reported significantly greater decreases in pain, depression, and fatigue, as well as greater improvements in mental health-related quality of life. A strength of the study is the larger sample size than Groessl’s earlier study. However, this larger sample size is tempered by the study’s focus on gender differences, with only 13 women participating. The third study was an RCT conducted with veterans with chronic low back pain [42]. Significant differences were seen between the yoga and the control groups for reduced pain at 6-month follow-up, as well as for reduced pain intensity at 12 weeks (immediately after completion of the yoga sessions) and at 6-month follow-up. This is the only study that used the gold standard, an RCT. The study is also notable in its focus on back pain. Multiple studies have found that back pain is one of the most common pain conditions [43,44]. To illustrate, data from the population-based National Health Interview Survey reveal that half of veterans reported back pain [6].

Groll and colleagues [39] carried out a study of yoga with active and retired members of the Canadian armed forces. Participants with PTSD reported significant pre/post differences, with decreased depression and anger and increased mental health-related quality of life after participating in the program. The PTSD group also exhibited a trend toward decreased pain. By contrast, findings for participants without PTSD were nonsignificant for all outcomes, including pain. A strength of the study is that it examined findings among study participants who had PTSD as well as those who did not. As has been stated, chronic pain is highly comorbid with PTSD [4,7,8,9,10]. Another strength is assessment of multiple outcomes that are comorbid with chronic pain, although the primary focus of the paper was on PTSD. A weakness is the small sample number of those who did not have PTSD (*n* = 14), which may explain the lack of findings for these participants. This study was conducted with Canadian participants, whereas the other studies reported here were U.S. samples.

King, Beehler, and Wade [45] studied a yoga intervention for older veterans who were cancer survivors. The study used a pre/post design. There were no significant pre/post changes for any outcomes: pain, anxiety, insomnia, fatigue, or PTSD. A strength of the study is its wide array of outcomes addressing comorbid conditions (anxiety, PTSD) and quality of life (insomnia, fatigue). Another strength is adaptation of yoga to the age of the participants (mean age 66 years) and their medical conditions. Specifically, the physical poses were completed in a chair, the pace of the intervention was slowed, and facilitators spoke loudly. A weakness of the study is its small sample size (*N* = 15), which likely precluded finding significant effects. 

Schulz-Heik’s study [41] compared yoga across two conditions: in person and via telehealth. Participants were not randomized to condition. Participants reported on whether yoga led to improved mental health and physical functioning. Only post-data were collected. There were no significant differences between the two conditions on any of the outcomes (back pain; headaches; upset stomach; constipation or diarrhea; sleep difficulties; low energy level; irritability; angry outbursts; difficulty concentrating; depression; anxiety; exaggerated startle response; and repeated, disturbing memories). More than 80% of participants reported decreased pain, depression, and anxiety and increased energy level. One weakness of the study is the wide variability in the number of sessions (range from 1 to more than 21 sessions). An additional weakness is that data were collected only after the yoga intervention. The study is innovative in its examination of yoga via telehealth. Given the long distances that some veterans must travel to a Veterans Affairs health care facility, this modality has the potential to increase access to yoga.

## 4. Discussion

Chronic pain is a serious problem in active duty and veteran populations and presents treatment challenges. A well-established body of literature underscores that rates of chronic pain among active duty and veteran populations are exceedingly high [5,6]. Chronic pain also affects quality of life, emotional functioning, and coping strategies. In addition, chronic pain negatively affects relationships and physical and social functioning [46,47]. Treating chronic pain is compounded by its high co-occurrence with PTSD [8]. Individuals with both chronic pain and PTSD have more frequent health problems, greater pain-related disability, higher ratings of pain, and increased functional impairment [48]. Further compounding the treatment of chronic pain is the use of opioids. Despite their widespread use, opioids have limited effectiveness and can lead to abuse or dependence [19]. 

The military is promoting the use of CIH strategies, including yoga, to address chronic pain. DoD’s Pain Management Task Force recommended the use of CIH modalities to treat chronic pain [27]. Research on CIH strategies is being conducted, but very little is known about the effectiveness of these approaches for chronic pain in military populations. The NIH’s National Center for Complementary and Integrative Health (NCCIH), the DoD, and the Department of Veterans Affairs have developed a partnership [49] that includes funding from both the NCCIH and the DoD for research on mind-body interventions, including yoga, to treat chronic pain. This partnership has the potential to move the field forward in determining the effectiveness of mind-body interventions with active duty soldiers and veterans in treating chronic pain. Research is particularly needed with younger OEF/IEF veterans who served in Afghanistan and Iraq, for whom the prevalence of severe pain is particularly high.

Research on the effectiveness of yoga with active duty and veteran populations to treat chronic pain is in its infancy. Our review identified only six studies, only one of which was an RCT. The remaining studies were pre/post (four studies) or post-only (one study) designs. Despite this limited literature base, these studies contain several strengths. In addition to pain, the studies assessed a wide array of outcomes. These measures consider conditions that co-occur with pain, such as insomnia [4,50] and PTSD [9]. Studies also included both sexes. One study looked at yoga delivered via telehealth, a modality that provides greater access than in-person administration. 

The current body of literature also has several limitations. Sample sizes were small, and all but one of the studies were conducted with veterans; the other was conducted with a combination of veterans and active duty soldiers. Two studies compared two yoga conditions (yoga in person or by telehealth; men and women). However, the small sample sizes in these studies likely precluded finding significant differences. In addition, in one study, participants could attend as many or as few classes as desired. With one exception, studies used uncontrolled designs.

## 5. Implications for Research and Practice

The studies in this review inform directions for future research. Particularly needed are RCTs with larger sample sizes to have sufficient statistical power to detect change. It is striking that we found no studies that included only active duty service members. Active duty service members likely experience and receive treatment for more recent chronic pain. As such, their response to yoga may differ from veterans whose chronic pain is not as recent. In addition, only one of the studies included OEF/IEF veterans. More than 2 million troops were deployed to Iraq and Afghanistan [51]; the estimated number of active duty service members is over 1 million, with an additional 801,000 in reserve components [52]. In addition, as noted, chronic pain is highly comorbid with PTSD [4,12]. It is possible that a more complex, trauma-informed yoga intervention is needed to reduce both chronic pain and PTSD, given that these conditions mutually maintain each other. Specifically, chronic pain may aggravate PTSD symptoms in terms of increased perceived pain, and disability. Similarly, the physiological and affective components of PTSD may amplify chronic pain symptoms [53]. We further recommend including measures of chronic pain in studies testing yoga with other conditions (e.g., PTSD) to increase the body of research on the effects of yoga.

A major practice consideration is integration of yoga in military settings. A recent report on CIH services in the military health system indicated that most military treatment facilities (MTFs) offered at least one type of CIH service [54]. Nonetheless, as noted previously, only 6.8% of active duty personnel reported using yoga in the last year [32]. Barriers to CIH service provision included a lack of available providers, support by the facility, provider awareness and interest, and space. We recommend that attention be given to the integration of yoga into MTFs, including increasing awareness among providers and incorporating yoga into chronic pain treatment protocols [55].

## 6. Conclusions

In conclusion, our literature review yielded only a small number of studies that tested the effects of yoga on pain in active duty or veteran populations. Sample sizes were small, and with one exception, studies used nonrandomized pre/post or post-only designs. Studies that have greater methodological rigor, such as RCTs, and that include a wide array of outcomes related to comorbid conditions are needed. Despite these limitations, this small body of literature sets the stage for future research with increased methodological rigor. Taken as a whole, results indicate that yoga shows promise for active duty and veteran personnel who experience pain. Consideration can be given to working with MTFs to integrate yoga into routine recommended care for chronic pain patients seeking treatment.

## Figures and Tables

**Table 1:** Sample Characteristics for Studies of Chronic Pain and Yoga in the Military.

Study	Participants	Mean Age	Sex	Race/Ethnicity	Marital Status	Employment
Groessl et al., 2008 [37]	VA patients with a diagnosis of chronic benign low back pain of at least 6 months’ duration and minimal use of narcotic medication	55 years	21% female	White 70%; African American 12%; Hispanic 12%; Asian/Pacific Islander 12%; other 3%	Married 52%; never married 21%; separated or divorced 24%; widowed 1%	Employed 39%; unemployed 3%; retired 37%; other 3%
Groessl et al., 2012 [38]	VA patients with a diagnosis of chronic benign low back pain of at least 6 months’ duration and minimal use of narcotic medication	Men, 56 years; women, 42 years	25% female	White 48%; African American 15%; Hispanic 13%; Asian/Pacific Islander 11%; other 2%	Married/partner 49%; widowed 2%; divorced/separated 30%; single 19%	Employed 36%; disabled 21%; retired 26%; unemployed 9%; other 8%
Groessl et al., 2017 [39]	Veterans with chronic low back pain for 6 months or more	53	26% female	White 53%; African American 19%; Hispanic 20%; Asian/Pacific Islander 5%; other 6%	Married 33%; single 24%; separated or divorced 39%; 3% widowed	Employed 32%; disabled 12%; unemployed 13%; retired or volunteer 20%; other 9%
Groll et al., 2016 [39]	Active and retired members of the Canadian armed forces who self-identified as having experienced at least one traumatic operational event	Not reported; most participants were 30–59 years old	29% female	Not provided	Single 8%; married/partner 66%; divorced 7%; widowed 4%; separated 13%	Full-time 69%; part-time 11%; on leave 2%; retired 16%
King et al., 2014 [40]	Older veterans diagnosed with cancer within the past 3 years	Not reported	13% female	White 93%; other race/ethnicities not reported	Not provided	Not provided
Schulz-Heik et al., 2017 [41]	Veterans: 48% Vietnam, 18% Desert Storm, 18% Operation Enduring Freedom/Iraqi Freedom, 14% multiple/other era, 2% Korean conflict	Not reported; plurality were Vietnam veterans	27% female	Not provided	Not provided	Not provided

Note. VA, Veterans Affairs.

**Table 2:** Yoga Intervention, Study Design, and Results of Studies of Chronic Pain and Yoga in the Military.

Study	Sample Size	Yoga Content	Duration and Frequency	Study Design	Dependent Variables	General Findings: Effectiveness
Groessl et al., 2008 [37]	33	Anusara yoga	10 weeks; one session per week with recommended at-home practice	Single group design Pre/post	Pain, depression, fatigue, quality of life (mental and physical)	Decreased pain, depression, fatigue; improved quality of life—mental health
Groessl et al., 2012 [38]	53	Anusara yoga	10 weeks; one session per week with recommended at-home practice	Two group design—men and women; both received yoga Pre/post	Pain, depression, fatigue, quality of life (mental and physical)	Women: greater improvements than men on depression, pain, fatigue, mental health-related quality of life
Groessl et al., 2017 [39]	150	Meditation, directed attention, postures, and home practice	12 weeks; 2 times per week; recommended home practice 15–20 min on non-session days	Randomized controlled trial; yoga and delayed yoga; assessments before and 6 weeks, 12 weeks, and 6 months after	Back-related disability, pain intensity, narcotic pain medication	Reduced back-related disability at 6-month follow-up; reduced pain intensity at 12 weeks and 6-month follow-up
Groll et al., 2016 [39]	45	Mindful yoga: breath work, meditation, mindful movements, guided resting practices, and gratitude	12 weeks; one session per week with recommended home practice	Single group design Pre/post	Depression, health, quality of life—physical and emotional, sleep, anxiety, anger, pain	Decreased depression, anger, pain (trend). Improved quality of life and sleep. Participants with PTSD showed greater improvements than those without PTSD.
King et al., 2014 [40]	15	8 modified yoga poses in combination with awareness, breathing, and relaxation exercises	8 weeks; two 75-min sessions per week with 15 min of recommended daily at-home practice at least 5 days per week	Single group design Pre/post	PTSD, anxiety, depression, fatigue, insomnia, pain	No statistically significant findings
Schulz-Heik et al., 2017 [41]	64	Meditation, intention setting, controlled breath practices, postures, synchronization of breath and movement, relaxation/meditation. Adapted for study population (use of chair; slow pacing).	Students not required to attend a specific number of sessions	Two group design—in-person and telehealth yoga Post only data	Back pain; headaches; upset stomach; constipation or diarrhea; sleep difficulties; energy level; irritability; angry outbursts; difficulty concentrating; depression; anxiety; exaggerated startle reflex; repeated, disturbing memories	No significant differences between in person and telehealth conditions. More than 80% of participants reported improvement in pain, energy level, depression, and anxiety.

Note. PTSD, posttraumatic stress disorder.

## References

[1] Clancy C. Statement of Dr. Carolyn Clancy, M.D. Interim Under Secretary for Health Veterans Health Administration (VHA) Department of Veterans Affairs (VA) before the Committee on Veterans’ Affairs United States Senate. https://www.veterans.senate.gov/imo/media/doc/VA%20Clancy%20Testimony%203.26.20151.pdf.

[2] Helmer D.A., Chandler H.K., Quigley K.S., Blatt M., Teichman R., Lange G. (2009). Chronic widespread pain, mental health, and physical role function in OEF/OIF veterans. Pain Med..

[3] Van Den Kerkhof E.G., Carley M.E., Hopman W.M., Ross-White A., Harrison M.B. (2014). Prevalence of chronic pain and related risk factors in military veterans: A systematic review. JBI Database Syst. Rev. Implement. Rep..

[4] Lew H.L., Otis J.D., Tun C., Kerns R.D., Clark M.E., Cifu D.X. (2009). Prevalence of chronic pain, posttraumatic stress disorder, and persistent postconcussive symptoms in OIF/OEF veterans: Polytrauma clinical triad. J. Rehabil. Res. Dev..

[5] Toblin R.L., Quartana P.J., Riviere L.A., Walper K.C., Hoge C.W. (2014). Chronic pain and opioid use in U.S. soldiers after combat deployment. JAMA Internal Med..

[6] Nahin R.L. (2017). Severe pain in veterans: The effect of age and sex, and comparisons with the general population. J. Pain.

[7] Bosco M.A., Murphy J.L., Clark M.E. (2013). Chronic pain and traumatic brain injury in OEF/OIF service members and Veterans. Headache.

[8] Gauntlett-Gilbert J., Wilson S. (2013). Veterans and chronic pain. Br. J. Pain.

[9] Otis J.D., Keane T.M., Kerns R.D. (2003). An examination of the relationship between chronic pain and post-traumatic stress disorder. J. Rehabil. Res. Dev..

[10] Clegg L.X., Arugay-Rittenberg D., Bullock E., McClafferty N., Penny R.R., Smith P. (2014). Healthcare Inspection—VA Patterns of Dispensing Take-Home Opioids and Monitoring Patients on Opioid Therapy.

[11] Alschuler K.N., Otis J.D. (2013). Significant others’ responses to pain in veterans with chronic pain and clinical levels of post-traumatic stress disorder symptomatology. Eur. J. Pain.

[12] McGeary D., Moore M., Vriend C.A., Peterson A.L., Gatchel R.J. (2011). The evaluation and treatment of comorbid pain and PTSD in a military setting: An overview. J. Clin. Psychol. Med. Settings.

[13] Alschuler K.N., Otis J.D. (2012). Coping strategies and beliefs about pain in veterans with comorbid chronic pain and significant levels of posttraumatic stress disorder symptoms. Eur. J. Pain.

[14] Shipherd J.C., Keyes M., Jovanovic T., Ready D.J., Baltzell D., Worley V., Gordon-Brown V., Hayslett C., Duncan E. (2007). Veterans seeking treatment for posttraumatic stress disorder: What about comorbid chronic pain?. J. Rehabil. Res. Dev..

[15] Pence P.G., Katz L.S., Huffman C., Cojucar G. (2014). Delivering integrative restoration-yoga nidra meditation (iRest(R)) to women with sexual trauma at a veteran’s medical center: A pilot study. Int. J. Yoga Therap..

[16] Staples J.K., Hamilton M.F., Uddo M. (2013). A yoga program for the symptoms of post-traumatic stress disorder in veterans. Mil. Med..

[17] Stoller C.C., Greuel J.H., Cimini L.S., Fowler M.S., Koomar J.A. (2012). Effects of sensory-enhanced yoga on symptoms of combat stress in deployed military personnel. Am. J. Occup. Ther..

[18] Furlan A.D., Sandoval J.A., Mailis-Gagnon A., Tunks E. (2006). Opioids for chronic noncancer pain: A meta-analysis of effectiveness and side effects. CMAJ.

[19] Ballantyne J.C., Shin N.S. (2008). Efficacy of opioids for chronic pain: A review of the evidence. Clin. J. Pain.

[20] Chou R., Turner J.A., Devine E.B., Hansen R.N., Sullivan S.D., Blazina I., Dana T., Bougatsos C., Deyo R.A. (2015). The effectiveness and risks of long-term opioid therapy for chronic pain: A systematic review for a National Institutes of Health Pathways to Prevention Workshop. Ann. Intern. Med..

[21] Trescot A.M., Glaser S.E., Hansen H., Benyamin R., Patel S., Manchikanti L. (2008). Effectiveness of opioids in the treatment of chronic non-cancer pain. Pain Phys..

[22] Department of the Army (2015). Health Promotion, Risk Reduction, and Suicide Prevention.

[23] Toblin R.L., Mack K.A., Perveen G., Paulozzi L.J. (2011). A population-based survey of chronic pain and its treatment with prescription drugs. Pain.

[24] The Opiod Therapy for Chronic Pain Work Group Clinical Practice Guideline for Opiod Therapy for Chronic Pain. https://www.healthquality.va.gov/guidelines/Pain/cot/VADoDOTCPG022717.pdf.

[25] Wilder C.M., Miller S.C., Tiffany E., Winhusen T., Winstanley E.L., Stein M.D. (2016). Risk factors for opioid overdose and awareness of overdose risk among veterans prescribed chronic opioids for addiction or pain. J. Addict. Dis..

[26] Seal K.H., Shi Y., Cohen G., Cohen B.E., Maguen S., Krebs E.E., Neylan T.C. (2012). Association of mental health disorders with prescription opioids and high-risk opioid use in US veterans of Iraq and Afghanistan. JAMA.

[27] Office of the Army Surgeon General Pain Management Task Force. Final Report. Providing a Standardized DoD and VHA Vision and Approach to Pain Management to Optimize the Care for Warriors and their Families. http://armymedicine.mil/Documents/Pain-Management-Task-Force.pdf.

[28] National Pain Strategy A Comprehensive Population Health-Level Strategy for Pain. https://iprcc.nih.gov/docs/DraftHHSNationalPainStrategy.pdf.

[29] U.S. Department of Veterans Affairs, Veterans Health Administration (2015). FY 2015 VHA Complementary & Integrative Health (CHI) Services (Formerly CAM).

[30] Department of Defense (2013). Yoga. The Continuum.

[31] Denneson L.M., Corson K., Dobscha S.K. (2011). Complementary and alternative medicine use among veterans with chronic noncancer pain. J. Rehabil. Res. Dev..

[32] Goertz C., Marriott B.P., Finch M.D., Bray R.M., Williams T.V., Hourani L.L., Hadden L.S., Colleran H.L., Jonas W.B. (2013). Military report more complementary and alternative medicine use than civilians. J. Altern. Complement. Med..

[33] Art of Living Foundation Sudarshan Kriya: Powerful Rhythmic Breathing Technique!. https://www.artofliving.org/sudarshan-kriya.

[34] Miller R. (2011). iRest at Ease: Guided Mediations for Active Duty Military, Veterans and Families [Audio Recording].

[35] Miller R. (2010). Yoga Nidra: A Meditative Practice for Deep Relaxation and Healing.

[36] Integrative Restoration Institute Overview of iRest in the Military. https://www.irest.us.

[37] Groessl E.J., Weingart K.R., Aschbacher K., Pada L., Baxi S. (2008). Yoga for veterans with chronic low-back pain. J. Altern. Complement. Med..

[38] Groessl E.J., Weingart K.R., Johnson N., Baxi S. (2012). The benefits of yoga for women veterans with chronic low back pain. J. Altern. Complement. Med..

[39] Groll D., Charbonneau D., Bélanger S., Senyshyn S. (2016). Yoga and Canadian Armed Forces members’ well-being: An analysis based on select physiological and psychological measures. J. Mil. Veteran Family Health.

[40] King K., Gosian J., Doherty K., Chapman J., Walsh C., Azar J.P., Danhauer S.C., Moye J. (2014). Implementing yoga therapy adapted for older veterans who are cancer survivors. Int. J. Yoga Therap..

[41] Schulz-Heik R.J., Meyer H., Mahoney L., Stanton M.V., Cho R.H., Moore-Downing D.P., Avery T.J., Lazzeroni L.C., Varni J.M., Collery L.M. (2017). Results from a clinical yoga program for veterans: Yoga via telehealth provides comparable satisfaction and health improvements to in-person yoga. BMC Complement. Altern. Med..

[42] Groessl E.J., Liu L., Chang D.G., Wetherell J.L., Bormann J.E., Atkinson J.H., Baxi S., Schmalzl L. (2017). Yoga for military veterans with chronic low back pain: A randomized clinical trial. Am. J. Prev. Med..

[43] Kang H.K., Mahan C.M., Lee K.Y., Magee C.A., Murphy F.M. (2000). Illnesses among United States veterans of the Gulf War: A population-based survey of 30,000 veterans. J. Occup. Environ. Med..

[44] Unwin C., Blatchley N., Coker W., Ferry S., Hotopf M., Hull L., Ismail K., Palmer I., David A., Wessely S. (1999). Health of UK servicemen who served in Persian Gulf War. Lancet.

[45] King P.R., Beehler G.P., Wade M.J. (2015). Self-reported pain and pain management strategies among veterans with traumatic brain injury: A pilot study. Mil. Med..

[46] Sanders S.H., Harden R.N., Vicente P.J. (2005). Evidence-based clinical practice guidelines for interdisciplinary rehabilitation of chronic nonmalignant pain syndrome patients. Pain Pract.

[47] Gatchel R.J., Peng Y.B., Peters M.L., Fuchs P.N., Turk D.C. (2007). The biopsychosocial approach to chronic pain: Scientific advances and future directions. Psychol. Bull..

[48] Hoge C.W., Terhakopian A., Castro C.A., Messer S.C., Engel C.C. (2007). Association of posttraumatic stress disorder with somatic symptoms, health care visits, and absenteeism among Iraq war veterans. Am. J. Psychiatry.

[49] NCCIH Council Working Group Strengthening Collaborations with the U.S. Department of Defense and U.S. Department of Veterans Affairs: Effectiveness Research on Mind and Body Interventions. https://nccih.nih.gov/sites/nccam.nih.gov/files/Council_Working_Group_Final_Report_2015-01-27.pdf.

[50] Tang N.K. (2008). Insomnia co-occurring with chronic pain: Clinical features, interaction, assessments and possible interventions. Rev. Pain.

[51] Hendricks A., Amara J., Hendricks A. (2013). OEF/OIF demographics compared to previous cohorts: Implications for medical issues. Military Medical Care: From Pre-Deployment to Post- Separation.

[52] U.S. Department of Defense Department of Defense (DoD) Releases Fiscal Year 2017 President’s Budget Proposal. https://www.defense.gov/News/News-Releases/News-Release-View/article/652687/department-of-defense-dod-releases-fiscal-year-2017-presidents-budget-proposal/.

[53] Sharp T.J., Harvey A.G. (2001). Chronic pain and posttraumatic stress disorder: Mutual maintenance?. Clin. Psychol. Rev..

[54] Herman P.M., Sorbero M.E., Sims-Columbia A.C. (2017). Complementary and Alternative Medicine in the Military Health System.

[55] Committee on Appropriations, U.S. Senate (2015). Improving military medicine’s management of pain. Department of Defense Appropriations Bill.

